# New Perspectives of Therapies in Osteogenesis Imperfecta—A Literature Review

**DOI:** 10.3390/jcm13041065

**Published:** 2024-02-13

**Authors:** Alexandru Dinulescu, Alexandru-Sorin Păsărică, Mădălina Carp, Andrei Dușcă, Irina Dijmărescu, Mirela Luminița Pavelescu, Daniela Păcurar, Alexandru Ulici

**Affiliations:** 1 Departament of Pediatrics and Department of Pediatric Orthopedics, “Carol Davila“ University of Medicine and Pharmacy, 020021 Bucharest, Romania; alexandru.dinulescu@drd.umfcd.ro (A.D.); madalina.carp@drd.umfcd.ro (M.C.); andrei.dusca@drd.umfcd.ro (A.D.); irina.dijmarescu@umfcd.ro (I.D.); mirela.pavelescu@umfcd.ro (M.L.P.); alexandru.ulici@umfcd.ro (A.U.); 2 Departament of Pediatrics and Department of Pediatric Orthopedics, Emergency Hospital for Children ‘’Grigore Alexandrescu’’, 011743 Bucharest, Romania; alexandru-sorin.pasarica@rez.umfcd.ro

**Keywords:** osteogenesis imperfecta, mesenchymal stem cells, sclerostin inhibition, anti-RANKL antibodies, gene therapy, recombinant PTH, anti-TGF-β antibodies, inhibition of eIF2α phosphatase enzymes

## Abstract

(1) Background: Osteogenesis imperfecta (OI) is a rare skeletal dysplasia characterized as a heterogeneous disorder group with well-defined phenotypic and genetic features that share uncommon bone fragility. The current treatment options, medical and orthopedic, are limited and not efficient enough to improve the low bone density, bone fragility, growth, and mobility of the affected individuals, creating the need for alternative therapeutic agents. (2) Methods: We searched the medical database to find papers regarding treatments for OI other than conventional ones. We included 45 publications. (3) Results: In reviewing the literature, eight new potential therapies for OI were identified, proving promising results in cells and animal models or in human practice, but further research is still needed. Bone marrow transplantation is a promising therapy in mice, adults, and children, decreasing the fracture rate with a beneficial effect on structural bone proprieties. Anti-RANKL antibodies generated controversial results related to the therapy schedule, from no change in the fracture rate to improvement in the bone mineral density resorption markers and bone formation, but with adverse effects related to hypercalcemia. Sclerostin inhibitors in murine models demonstrated an increase in the bone formation rate and trabecular cortical bone mass, and a few human studies showed an increase in biomarkers and BMD and the downregulation of resorption markers. Recombinant human parathormone and TGF-β generated good results in human studies by increasing BMD, depending on the type of OI. Gene therapy, 4-phenylbutiric acid, and inhibition of eIF2α phosphatase enzymes have only been studied in cell cultures and animal models, with promising results. (4) Conclusions: This paper focuses on eight potential therapies for OI, but there is not yet enough data for a new, generally accepted treatment. Most of them showed promising results, but further research is needed, especially in the pediatric field.

## 1. Introduction

Osteogenesis imperfecta (OI), also called brittle bone disease, Lobstein disease, or Vrolik syndrome, is a rare genetic disorder of the connective tissues characterized by skeletal dysplasia with bone fragility, caused by an abnormality in the metabolism of type I collagen, with a reported incidence of 1 in 15,000 to 20,000 births [1,2,3].

### 1.1. History

The first case of OI was reported by the French priest Melabranche in the 17th century (1674): a 20-year-old male with an intellectual disability and multiple bone fractures. A Swedish surgeon, Olaus Jakob Ekman, was the first to scientifically describe OI in the 18th century (1788), but the diagnosis of osteomalacia was considered for all his patients. In the 19th century, more features of the disease were described. Edmond Axman was the first, in 1831, to highlight the four major characteristics of OI: frail body, fragility of the bones, hypermobility with easy dislocation of the joints, and blue sclerae. Johann Lobstein identified, in 1833, the hereditary nature of the disease, but the term “osteogenesis imperfecta” was first introduced in 1849 by the Dutch professor Willem Vrolik. In the 20th century (1979), Sillence, Senn, and Danks proposed the most widely used classification of the disease, the Sillence classification [1,4].

### 1.2. Pathophysiology

Most patients (85%) with OI carry an autosomal dominant (AD) mutation in the genes that encode the production of type I collagen (*COL1A1* and *COL1A2*). There are two major mutations in these genes associated with either a structural or quantitative abnormality in the synthesis or processing of type I collagen. Also, autosomal recessive (AR) and X-linked variants of the disease have been described, affecting other genes [2,3,5,6,7].

### 1.3. Classification

The first and most widely used classification of OI was published in 1978 by Sillence. Over the years, the nosology of this disease has evolved with the identification of new genes and new mutations. The latest accepted classification was proposed by Botor et al. in 2021 and adapted, as shown in Table 1 [4,7,8].

**Table 1:** Classification of OI adapted by Botor et al. [7].

Type of OI	Mode of Inheritance	Mutated Genes	Clinical Characteristics
I	AD	*COL1A1* *COL1A2*	Mild form with increased bone fragility Non-deforming Normal stature Blue-gray sclerae Hearing-impaired
II	AD	*COL1A1* *COL1A2*	Perinatal death
	AR	*CRTAP* *P3H1*	Severe to lethal forms
III	AD	*COL1A1* *COL1A2* *IFITM5*	Progressive deformity
			Mild-to-moderate form
	AR	*SERPINF1* (type VI in the previous classifications)	Moderate to severe form; healthy at birth; no fracture until 6 months of age
		*CRTAP* *P3H1* *SERPINH1*	Severe to lethal forms
		*WNT1*	Moderate to severe form
		*BMP1*	Fewer than 20 individuals were diagnosed with this mild-to-severe form; increased bone mineral density (BMD)
		*FKBP10*	Broad spectrum of disease includes a lethal form of OI previously classified as type IX, Kuskokwim syndrome, and Bruck syndrome
IV	AD	*COL1A1* *COL1A2*	Moderately deforming
		*WNT1*	Moderately severe
		*IFITM5*	Mild-to-moderate form
	AR	*FKBP10*	Progressive form, deforming
		*SP7*	Moderate form
V	AD	*IFITM5*	Moderately deforming; calcification of interosseous membranes

### 1.4. Current Treatment of OI

The current treatment of OI varies with the age, severity, and functional status of patients. Therapeutic options include medical management with several pharmacological resources and orthopedic therapy.

#### 1.4.1. Medical Management or Pharmacological Treatment

The current gold standard in OI treatment is considered the use of bisphosphonates. Bisphosphonates are anti-resorptive drugs that inhibit osteoclasts’ activity and increase bone volume. The goal of bisphosphonate therapy is to counteract the high cellular turnover status, and although new bone is still made of poor-quality collagen, the increase in bone volume may be beneficial despite its impaired quality. It has been proven that they increase BMD, but the effect on fracture reduction is inconclusive. Also, they are most effective in the first year of treatment [3,5,6,7,9,10].

The most studied and used bisphosphonate in children is Pamidronate. Glorieux et al. published, in 1998, the first study with Pamidronate in children with OI, using a dose of 1.5–3 mg/kg for 3 consecutive days in 30 children, with repeated administrations at 4–6 months over 5 years (1992–1997). Ralston et al. published a systematic review in 2020, in which they claim to have found over 150 studies regarding the use of Pamidronate in OI that support Glorieux’s results of increasing BMD, improving symptoms, and reducing fracture rates. In our review of the literature focusing on Pamidronate usage in children with OI, we found different doses and intervals of administration without a clear consensus [11,12,13,14,15].

#### 1.4.2. Orthopedic Treatment

Surgical treatment is indicated in the case of fractures and for surgical correction of long bone deformities, using telescopic rods that extend during growth [5].

### 1.5. Aim and Scope

Bisphosphonate therapy, the current gold standard of treatment in OI, is well studied, but its efficacy is not fulfilling. In this review paper, we intended to identify in the scientific literature other potential therapies for OI that have been studied or are currently being studied.

The scope of this research was to review experimental or lesser-known therapies for OI supported by the scientific literature. A comparison among different management strategies for therapeutic efficacy is difficult to conduct and was not the aim of this current review. Analyzed studies were most frequently based on a small number of patients, and even case reports were included. Also, the considered studies included patients of different ages and with varying types of diseases; most of them had undergone failed previous therapies, but there were also some who had not been treated, with multiple complications.

## 2. Materials and Methods

### 2.1. Search Strategies

We reviewed the literature from the following medical databases: PubMed and The Cochrane Central Register of Controlled Trials (CENTRAL). We used the following keywords: osteogenesis imperfecta, brittle bones disease, Lobstein disease, and Vrolik syndrome, and were associated with keywords such as mesenchymal stem cells, MSCs, fetal mesenchymal stem cells, bone marrow mesenchymal stem cells, Denosumab, RANKL inhibitors, Salubrinal, Sclerostin inhibition, Sclerostin antibodies, Scl-Ab, romosozumab, Teriparatide, 4-phenylbutyric acid, 4-PBA, Anti-TGF-β Antibodies, Fresolimumab, gene therapy in OI, RNA interference, RNAi, small interfering RNAs, and siRNA.

### 2.2. Inclusion Criteria

We included in the selection animal model studies, cell studies, case reports, case series, and original articles written in English from January 1990 to November 2023, that covered treatment of OI other than bisphosphonates.

We selected articles that contained treatments for OI other than the conventional ones with bisphosphonates.

### 2.3. Data Extraction

Based on the keyword search, we found 2244 publications. We excluded 2201 of the articles, either because the inclusion criteria were not met, because duplicates were identified, or because the respective articles were not written in English. We included, after the selection, 45 publications. Of these 45 publications, 16 were animal model studies, 9 were cell studies and 20 of them included human research (14 of these were in children).

**Figure 1:**
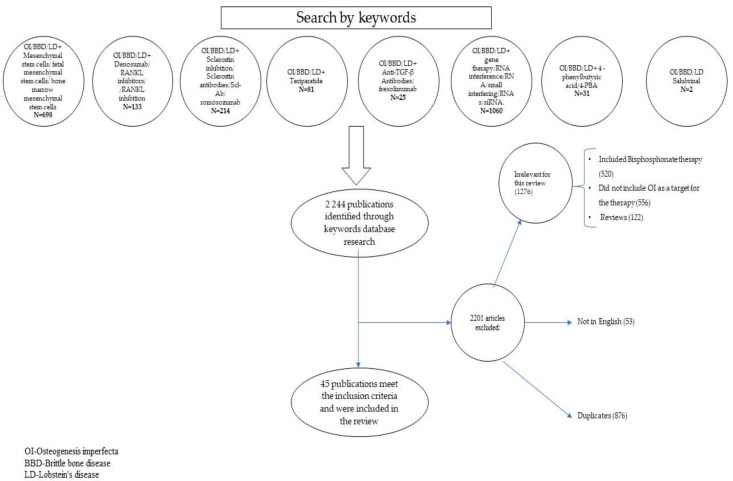
Data extraction.

The 45 selected publications were grouped by the type of treatments applied. We identified 15 studies about the mesenchymal stem cells (MSCs), 12 papers on sclerostin inhibition, nine types of research about anti-RANKL antibodies, 7 studies on gene therapy, and another 7 on 4-phenylbutyric acid (4-PBA), 2 papers about recombinant human parathyroid hormone (PTH), 1 study on anti-transforming growth factor βeta (TGF-β) antibodies and 1 about the inhibition of eukaryotic initiation factor-2α (eIF2α) phosphatase enzymes. The data extraction is found in Figure 1.

The studies included in this research are selected from peer-reviewed journals, strengthening the quality of the research. All publications are from journals indexed in Scopus, PubMed, SCImago, or Web of Science (Clarivate).

## 3. Discussions

### 3.1. Mesenchymal Stem Cells (MSCs) (Table 2)

Transplantation of bone marrow is being investigated as a promising therapy in OI in studies performed on mice and humans, both adults and children [7,10,11,16].

#### 3.1.1. Mechanism of Action

Adult stem cells are present in many tissues, but their number decreases with age, and the ones found in the bone marrow have the highest potential in terms of multi-lineage. MSCs are multi-potent stem cells that have the ability of self-renewal and proliferation, and can differentiate into multi-lineage cell types, including osteoblasts. These stem cells have the capacity to migrate into injury sites and secrete cytokines, chemokines, and growth factors useful for the regeneration of the tissue. For these reasons, MSCs become a source of continual renewal of the bone cells and production of healthy collagen and can attenuate genetic disorders of the bone. Immunomodulatory proprieties of the MSCs decrease the risk of immune rejection; therefore, allogenic transplants would not need immunosuppression of the host. Most MSCs used are from bone marrow cells; another source of the stem cells is fetal MSCs, which are less immunogenetic and have an increased proliferative, anti-inflammatory, and homing capacity than their adult counterparts. Guillot et al. published, in 2008, a study that compared human mesenchymal fetal stem cells (hfMSCs) and bone marrow mesenchymal stem cells (BMSCs) and noticed that the fetal cells had a higher level of osteogenic upregulation and produced more calcium, both in vitro and in vivo (murine) [17,18,19,20,21,22,23,24,25,26].

#### 3.1.2. Murine Studies

The studies performed on mice with OI are promising, with a significant increase in bone collagen and mineralization, improving the bone structure and reducing fracture incidence [25,27,28,29,30]. Battle and Co. published a 2021 meta-analysis of 10 studies about the MSCs’ efficacy in mouse models with OI and noticed an increase in the mechanical and structural bone proprieties and a decrease in fracture rates. The same meta-analysis reviewed data about cell engraftment in five studies and reported a low benefit [22].

#### 3.1.3. Human Studies

##### Bone Marrow MSCs (BMSCs)

The first trial of MCSs, which used BMSC therapy in children with OI, was published in 1999 by Horwitz et al. and reported an increase in BMD and growth velocity and the diminishing of fracture rates in three children with OI type III that received BMCSs from their siblings. Two years later, they published a follow-up study comparing the results for the three treated children with two control patients and reported a decline in the growth rate or a plateau phase in time; however, the BMD continued to increase at a similar rate in healthy children [31,32].

In 2021, Infante et al. published the TERCELOI clinical trial, conducted over a 2.5-year period, including two children with moderate and severe OI forms with repetitive infusions of their siblings’ BMSCs. Both patients showed an increase in BMD (bone volume to the total volume ratio) (BV/TV)), a decrease in fracture rates, and chronic pain. Also, the benefits were observed at a 2-year follow-up after halting the therapy [33].

##### Fetal MSCs

Götherström et al. published a 2013 study that included two infants with severe OI transplanted with hfMSCs pre- and postnatal, reporting promising results in their BMDs, motility, and fracture rates [34,35].

The trial, Boost Brittle Bones before birth (BOOSTB4), was conducted in Sweden at the Karolinska Institute between 1 January 2016 and 31 December 2022, with administration of hfMSCs pre- and postnatal in children with OI type III and type IV, without published results; however, Lindgren et al. reported no complications with the preliminary results on 17 participants receiving 1 to 4 doses of MSCs. The efficacy was yet to be evaluated [36,37].

Overall, the MSCs in OI look promising. These studies show potential in increasing the BMD and reducing the fracture rates in murine and human studies. Nevertheless, there are some limitations in these studies, like the fact that cells deteriorate under standard conditions, needing to be cryopreserved in special conditions, and the fact that some implications of MSCs in cancers and in worsening bacterial infections have been reported [27].

**Table 2:** Studies of MSCs transplantation in OI.

Reference	Methods	Comments
Horwitz et al. 1999, 2001 [31,32]	Case series: 3 children with OI type III; duration: 2 years	They enrolled 3 children with OI type III and intravenously administered BMSCs from siblings, with reported improvements in bone density and growth velocity and reduced fracture frequency in the first 3 months. Two years later, they published a follow-up study comparing the evolution of the 3 children treated with 2 control patients. The growth rate declined or reached a plateau phase in time, but the BMD continued to increase at a similar rate to healthy children. No complications were reported.
Götherström et al. 2005, 2013 [34,35]	Case series: 2 fetuses with OI (type III and IV); duration: 10 years and 1 year	The study included OI patients transplanted with hfMSCs pre- and postnatal: - A 32-week female fetus with severe OI phenotype underwent a prenatal MSCs transplantation; she had 3 fractures and no complications of pain in the first 2 years of life, and the psychomotor development was normal but small for her age (−5 SD). Between 2 and 8 years old, she suffered multiple complications and was transplanted again with hfMSCs. After the postnatal transplantation, she did not develop any new fractures in 2 years, and her linear growth followed the previous one; also, her walking improved, and she was able to play sports. - A female fetus with OI type IV was transplanted prenatal at 31 weeks of gestation (born at 38 weeks of gestation). Over the first 12 months, she had no fractures, and she grew along her centiles (third centile for length). After 12 months, her linear growth was impaired, so she had a postnatal transplant of hfMSCc at 19 months. Growth resumed along her centiles, and she began to walk. No complications were reported.
Infante et al. 2021 [33]	Clinical trial, phase 1, 2 children with OI	Two patients, a 6-year-old boy with a severe form of OI and an 8-year-old girl with a moderate form of OI, duration of 2.5 years. Both patients showed an increase in BMD and BV/TV, a decrease in fracture rates, and chronic pain. Benefits were observed after a 2-year follow-up visit after the ending of the therapy. No complications were reported.
Battle et al. 2021 [22]	Meta-analysis of animal studies, control trials in OI mice models of stem cell therapy period: 13 years	Ten studies were reviewed. The authors found an increase in mechanical proprieties like maximum load (*p* = 0.02), a decrease in fracture incidence (*p* < 0.00001), and a beneficial effect on structural proprieties: cortical thickness and BV/TV, but without statistical significance (*p* = 0.4; respectively, *p* = 0.31). Also, the meta-analysis had data about cell engraftment in 5 studies with poor results.

### 3.2. Anti-RANKL Antibody (Table 3)

Denosumab is a human monoclonal antibody with bone anti-resorptive effects, approved for osteoporosis in postmenopausal patients in 2010, as well as for other bone diseases such as giant cell tumors or bone metastases. In children, the pharmacological proprieties are unknown, but they could be used in some diseases, one of them being brittle bone disease [38,39,40].

#### 3.2.1. Mechanism of Action

Denosumab is an IgG2 human monoclonal antibody that reduces bone resorption and increases bone mass. It binds to the receptor activator of NF kappa B ligand (RANKL) and blocks its binding to the receptor (RANK). By binding on RANK, RANKL promotes osteoclast activity and increases bone resorption. This binding is normally blocked by soluble osteoprotegerin (OPG), a member of the tumor necrosis factor (TNF) receptor family. Denosumab mimics the anti-resorptive effect of OPG by blocking RANKL [40,41,42].

#### 3.2.2. Human Studies

In 2012, Semler et al. published the first study of Denosumab use in OI. Four children with OI type VI were included and received Denosumab for 24 months, with an increase in BMD and improvement in their pain. One patient had mild hypocalcemia [43].

In 2014, an article reported two cases of OI children treated with Denosumab with improvements in their metaphyseal density on X-rays [44].

Hoyer-Kuhn et al. published, in 2016, a 48-week prospective trial on 10 children with OI who received four doses of Denosumab. The authors reported four fractures within this period without any beneficial effect on bone pain but with improvements in their BMD and height during this time. The X-ray showed new bone formation. The fracture frequency was not analyzed. In 2019, they published a follow-up study, with only 8 of those 10 patients included, who continued to receive Denosumab. The BMD had a significant reduction during the first follow-up year, but at the end of the follow-up, it was still higher than at the start of the trial. Vertebral shape improved further during the follow-up. Growth was not influenced, and mobility was not significantly improved. The mean calcium levels decreased in all patients, and calcium excretion on urinary spots increased; however, only one patient presented symptomatic hypercalciuria with urolithiasis [45,46].

In 2016, Ward et al. reported a case of a 23-month-old male patient with OI type VI treated with Denosumab. The results were disappointing, with a high fracture rate, no change in mineralization on a bone sample, and a high number of osteoclasts in trabecular bone [47].

In 2017, Uehara et al. published a study that included three female patients with OI type I: two adults and one adolescent who received Denosumab. They all had an improvement in their BMD during treatment, resorption markers, and bone formation, and no fracture occurred in any of them [48].

Trejo et al. published an article in 2018 about four children with OI type VI who received treatment with Denosumab. Their spinal BMD increased, but after an interval between administrations was increased at 6 months, the results were not maintained. All of them developed hypercalciuria in time, and two of them presented hypercalcemia [49].

Another study, published in 2018 by Kobayashi et al., analyzed eight patients with OI type I (six adults and two children) receiving Denosumab. Their BMD generally increased in all patients and the fracture rates and bone turnover markers decreased in most of the patients. They reported no hypocalcemia [50].

In 2019, Maldonado et al. published a case report of a 9-year-old girl with OI type IV treated with Denosumab and with a history of cerebral palsy and epilepsy. She had a decrease in bone resorption, an increase in quality of life, and a decrease in the fracture rate. Hypercalcemia was reported during treatment administration [51].

We only found case reports and two prospective cohort studies involving this therapy, therefore lacking high quality and strength of evidence. Of what we found, publications generally reported an increase in the BMD. In almost all cases, hypocalcemia is reported—this side effect of Denosumab is known. Serum calcium levels must be monitored [38,40,52].

**Table 3:** Studies of Anti-RANKL antibody (Denosumab) on OI.

Reference	Methods	Comments
Semler et al. 2012 [43]	Case reports: 4 children with OI type VI (AR-SERPINF1); duration: 24 months	All of the subjects who were previously treated with bisphosphonates without response received Denosumab (1 mg/kg) every 3 months for 24 months. An increase in BMD and improvement in pain were noticed. One patient had mild hypocalcemia.
Hoyer-Kuhn et al. 2014 [44]	Case reports: 2 children with OI (*COL1A1/A2*); duration: 36 weeks	They report 2 cases of children with poor responses to bisphosphonates for 4 years, who received 1 mg/kg Denosumab every 12 weeks (1 of them received 1 dose and 1 of them 3 doses). After treatment, an increase in metaphyseal density on X-rays was reported, with no complications.
Ward et al. 2016 [47]	Case report: 23-month-old male patient with OI type VI; duration: 12 months	A 23-month-old male child with OI type VI, previously treated with 2 doses of Zolendronate at 4 months apart, was inefficient (the same rate of bone fractures, 3 fractures in 6 months). He received Denosumab, 1 mg/kg every 3 months for 12 months. The results were poor, the fracture rate continued to be high, and the bone sample obtained after 5 doses of treatment at 15 months showed no change in mineralization, with an increased number of osteoclasts in trabecular bone. No complications were reported.
Hoyer-Kuhn et al. 2016 [45]	Prospective, single-arm, phase-2 trial: 10 children with OI, 8 type I and 2 type III; duration: 48 weeks (36 weeks administration and 12 weeks follow-up)	Ten children with OI who received at least 2 years of bisphosphonate, calcium, and vitamin D were included and treated with 4 doses of Denosumab (1 mg/kg every 12 weeks). An increase in the mean height was noticed but without a significant change in the z-score (*p* = 0.70). Four fractures occurred during this period. The treatment did not influence bone pain (*p* = 0.70) or mobility (*p* = 0.15), but the lumbar spine bone mineral density (LS-BMD) increased from −2.23 ± 2.03 to −1.27 ± 2.37 (*p* = 0.0006). Two patients reported arthralgia, and 1 child reported mild hypocalcemia.
Uehara et al. 2017 [48]	Case reports: 3 females with OI type I, 2 adults (42 and 40 years), 1 adolescent of 14 years; duration: 30 months	All 3 patients were treated with 5 doses of Denosumab (1 dose every 6 months). The results consist of the enhancement of BMD during treatment, improved resorption markers and bone formation, and no new fractures. The adolescent had an increase in height of 2 cm during treatment. No complications were reported.
Trejo et al. 2018 [49]	Case reports: 4 children with OI type V; duration variable from 1.3 years to 3.5 years.	The patients with bisphosphonate treatment failure received Denosumab 1 mg/kg every 3 months. - A 9-year-old boy with homozygous stop mutation in SERPINF1 received the treatment for 3.5 years with an increase in bone resorption markers carboxy-terminal collagen crosslinks (CTX) but developed hypercalciuria. - A 3.6-year-old boy with compound heterozygous SERPINF1 mutations received 5 doses of Denosumab, one dose every 3 months, with an increase in CTX, an increase in LS-BMD, and after respacing the doses at 6-month intervals, the BMD dropped, and the decision of administration of Denosumab every 3 months was taken with the increase in BMD. No complications were reported. - A 2.7-year-old girl with homozygous splice mutation in SERPINF1 received Denosumab, with an increase in the BMD (from −3.5 to −0.6), an increase in CTX and marked hypercalciuria and hypercalcemia. - A 1.9-year-old boy with homozygous splice mutation in SERPINF1 received their first Denosumab, one dose every 3 months, subsequently every 6 months, but after a widening of the interval, the BMD decreased considerably. One dose was recommended every 2 months, and the BMD increased. At age 4.6 years, hypercalcemia, hypercalciuria, and nephrocalcinosis were reported. The treatment increased spinal BMD, but not if the interval of administration was 6 months; as side effects, complications related to calcium levels were frequent.
Kobayashi et al. 2018 [50]	Case series: 8 OI type I patients (5 adults, 3 children); duration: 54 months.	These patients received 60 mg of Denosumab every 6 months (from 1 to 9 doses), and calcium and cholecalciferol. The BMD generally increased in all patients and the fracture rate and bone turnover markers decreased in most of the patients. No complications were reported.
Maldonado et al. 2019 [51]	Case report: a 9-year-old girl with OI type IV, cerebral palsy, and epilepsy; duration: 18 months	The child had a history of treatment with Pamindronate and Zolendronate with 40 fractures. She started Denosumab (3 doses of 60 mg, a dose every 6 months) with a decrease in bone resorption and an increase in quality of life; no fracture during the Denosumab treatment time, but hypercalcemia was reported.
Hoyer-Kuhn et al. 2019 [46]	Prospective cohort study: 10 children with OI, 8 with type I, and 2 with type III; duration: 48 weeks treatment and 12 months follow-up	Ten children with OI received Denosumab at a mean interval of 20.33 weeks for 48 weeks (4 doses). BMDs had a significant reduction during the first follow-up year, but at the end of the follow-up, it was still higher than at the start of the trial. Vertebral shape improved further in the follow-up. Growth was not influenced, and mobility was not significantly different. As side effects, a decrease in the mean of serum calcium levels (*p* = 0.00039) and, for one child, symptomatic hypercalciuria with urolithiasis were reported in the first year of follow-up.

### 3.3. Sclerostin Inhibition (Table 4)

#### 3.3.1. Mechanism of Action

Sclerostin is a glycoprotein secreted by osteocytes that acts as a negative regulator of osteoblast differentiation, bone formation, and mineralization, therefore blocking its activity. It may show promise in OI, its efficiency being demonstrated in other bone mineral deficiencies, such as osteoporosis in postmenopausal women. Some studies have also explored the use of sclerostin as a bone turnover marker in some metabolic bone diseases, including OI, with increased levels of sclerostin reported, while others reported normal levels of OI [53,54,55,56,57].

#### 3.3.2. Animal Studies

A study by Sinder et al., published in 2013, researched the use of sclerostin antibodies (Scl-Ab) in the *Brtl/+* mouse model of OI. They reported an anabolic effect on osteoblasts, with an increase in mechanical bone proprieties [58].

Jacobsen et al. published a study in February 2014 using Scl-Ab therapy in an OI-type IV mouse model. They reported an increased bone mass and strength in the treated mice compared with the control group [59].

Another study that used Scl-Ab on adult mice with OI was published by Sinder et al. in May 2014, in which Scl-Ab was administrated for 6 months to *Brtl/+* mice, and after therapy, an increase in the bone formation rate, trabecular cortical bone mass, and mechanical proprieties were reported [60].

In September 2014, Roschger et al. proposed a study about Scl-Ab in a model with severe OI, *Col1a1Jrt/+* mice, including growing and adult mice. The authors found no significant changes in bone formation or resorption markers but reported a higher trabecular volume and cortical thickness in growing mice and no changes in the adult ones, concluding that this treatment was less effective in the severe OI mouse model [61].

Sinder et al. published a study in 2015 in which Scl-Ab was used on normal mice and rapidly grown mice with OI (*Brtl/+*), and reported an increased bone cortical mass and improvements in mechanical strength on those treated with Scl-Ab, with a stronger effect in the wild types. In 2016, Sinder et al. published another study in which they used Scl-Ab in normal mice and mice with OI (*Brtl/+*) and reported an increase in the mineral matrix in the adult model, but without affecting the elastic module [62,63].

A study published by Grafe et al. in 2016 studied the effect of Scl-Ab on young recessive OI mice *Crtap−/−.* Compared with the control group, the Scl-Ab mice showed improvement in bone mass, bone formation, parameters of strength, and trabecular microarchitecture [64].

Cardinal et al., in 2019, published a study on their OI type III mice model, in which Scl-Ab reduced long bone fractures and increased the BMD and biomechanical strength of bones [65].

In 2022, Wang et al. published a study that only used loop 3-specific antibodies of sclerostin (aptscl56) in mice with OI. Because romosozumab, an Scl-Ab used in postmenopausal women for osteoporosis, had a related increased cardiovascular risk, the authors theorized that the protective cardiovascular effect of sclerotin was particular to loop 3 inhibition, while the cardiovascular risk was increased by the inhibition of loop 1 and loop 2. A conjugation of aptck56 with PEG40k raises the stability and the half-life, resulting in PEG40k-aptscl56 (Apc001PE). Apc001PE promoted the formation of the bone without a higher cardiovascular risk [66].

#### 3.3.3. Human Studies

Glorieux et al. reported, in 2017, a phase 2a trial of Scl-Ab (BPS804) in adults with moderate OI. The authors concluded that the antibody increased the BMD, reduced resorption, and stimulated bone formation [55].

In 2021, Uehara et al. published a case report of a 64-year-old severe osteoporotic man with OI type I, treated with romosozumab for 12 months with an increase in his BMD, bone formation markers, and a decrease in his resorption markers [67].

Another case report of romosozumab use in OI was published by Dattagupta et al. in 2023. The subject was a 52-year-old woman with type I OI who underwent alendronate therapy for almost 1 year—8 years before this article was published—without an increase in her BMD. She received romosozumab for 1 year, and an increase in BMD was reported [68].

The reports are generally favorable, with an increase in the BMD. As for limits, we only found three studies that involve humans, none of these studies focusing on children, and two of them being case reports.

**Table 4:** Studies of sclerostin inhibition in OI.

Reference	Methods	Comments
Sinder et al. 2013 [58]	Animal study: 8-week-old *Brtl/+* mice; duration: 2 weeks	Randomized 8-week-old Brtl/+ mice and WT mice received 25 mg/kg Scl-Ab twice a week for 2 weeks. The body length was unchanged, but increased anabolic responses in the treated mice and an increase in BV/TV, cortical formation, and mechanical proprieties were reported.
Jacobsen et al. 2014 [59]	Animal study: 6-week-old *Col1a2^+/p. G610C^* mice, model human OI type IV; duration: 6 weeks	Randomized OI type IV 6-week-old mice models received 25 mg/kg Scl-Ab twice a week for 6 weeks. At the end of the study, a significant rise in BV/TV, BMD, and bone strength was present in the treated mice compared with the OI model controller. The bone parameters were similar to or even greater than the wild-type mice without treatment. No complications were reported.
Sinder et al. 2014 [60]	Animal study: 6-month-old *Brtl/+* mice, model human OI type IV; duration: 5 weeks	Randomized OI type IV 6-month-old mice models received 25 mg/kg Scl-Ab twice a week for 5 weeks. An increase in bone formation rate and trabecular cortical bone mass were reported. Also, the mechanical tests and the strength and stiffness of the femoral bones increased. No complications were reported.
Roschger et al. 2014 [61]	Animal study: growing (4 weeks) and adult (20 weeks) *Col1a1^Jrt^/+* mice, pediatric and adult model of severe OI; duration: 4 weeks	Growing (4 weeks) and adult (20 weeks) *Col1a1^Jrt^/+* mice, pediatric and adult models of severe OI, received 100 mg/kg Scl-Ab once a week for 4 weeks, with no significant changes in bone formation or resorption markers but higher trabecular volume and cortical thickness in growing mice, and no changes in the adult ones. The additional cortical formation reported in younger mice was located on the endocortical surface, with a minor effect on bone resistance to bending and the mechanical testing did not reveal a positive change in both growing and old OI mice. The authors concluded that this treatment was less effective in severe OI mouse models, pediatric or adult. No complications were reported.
Sinder et al. 2015 [62]	Animal study: 3-week-old *Brtl/+* mice, model human OI type IV; duration: 5 weeks	Randomized OI type IV 3-week-old mice models received 25 mg/kg Scl-Ab twice a week for 5 weeks, and an increase in bone formation, bone cortical mass, and improvements in mechanical strength in the models treated with Scl-Ab were noticed without complications.
Sinder et al. 2016 [63]	Animal study: growing (3 weeks) and adult (6 months) *Brtl/+* mice, pediatric and adult model of human OI type IV; duration: 5 weeks	Growing (3 weeks) and adult (6 months) Brtl/+ mice, pediatric and adult models of human OI type IV, received 25 mg/kg Scl-Ab twice a week for 5 weeks. The collected bone samples from the right femur showed the mineral matrix enhanced in the adult model but not significantly in the pediatric one. The elastic module was not increased in any model. No complications were reported.
Grafe et al. 2016 [64]	Animal study: 1 and 6 weeks *Crtap−/−* mice, pediatric and young adult models of recessive OI; duration: 6 and 7 weeks	Crtap−/− mice models for recessive OI were treated with 25 mg/kg Scl-Ab twice a week for 6 weeks in the young adult model (6 weeks old) and for 7 weeks in the pediatric ones. The improvement in bone mass, bone formation, parameters of strength, and trabecular microarchitecture were reported, and there was a decrease in the number of osteoclasts without complications.
Glorieux et al. 2017 [55]	Randomized, controlled human phase 2 study: 13 adults with moderate OI completed the study. They defined moderate OI as types I, III, or IV with a history of at least two fractures. In the control group (5), 2 had OI type I, and 3 had OI type III/IV. One of them was lost to follow-up and did not receive a DXA scan in the end. They do not specify what type this subject was. In the treatment group (9), 4 of them were type I, and 5 were type III/IV; duration: 21 weeks	The treatment group received 3 doses of Scl-Ab (BPS804) (day 1: 5 mg/kg; day 15: 10 mg/kg; day 29: 20 mg/kg) and was followed for 14 weeks. The biomarkers were significantly increased, and downregulation of the bone resorption marker (CTX-1) in the study drug group was noticed, compared to the control group (44% vs. 7%). The lumbar spine BMD (LsBMD) on day 141 was increased by 4% in the Scl-Ab group compared with only a 1% increase in the reference group. No serious adverse events were reported in the BPS804 treatment group (no abnormal calcium blood levels or other laboratory test abnormalities). Three fractures in the treatment group (2 on day 2 and 1 on day 48) were present, and none in the control group. The Scl-Ab increased the BMD, reduced resorption, and stimulated bone formation, and thus, the study opens the possibility of a phase 3 trial study.
Cardinal et al. 2019 [65]	Animal study: 5-week-old *B6C3Fe a/a-Col1a2^oim^/J* mice, model for OI type III; duration: 9 weeks	Wild-type and old mice were treated with Scl-Ab for 9 weeks, 50 mg/kg once a week, with an increase in BMD, ultimate load, stiffness, plastic energy, and elastic modulus, and significantly reduced long bone fractures, with no complications.
Uehara et al. 2021 [67]	Case report: 64-year-old severe osteoporotic man with OI type I, previously treated with alendronate for 1 year, 8 years before this article; duration: 12 months	The subject received romosozumab, one dose of 210 mg monthly for 12 months, and vitamin D (eldecalcitol). The evaluation of BDM at 6 and 12 months of treatment revealed an improvement in BMD and in the turnover markers, with no fracture or other complications during the study time.
Wang et al. 2022 [66]	Animal study: *Col1a2+/G610C.ApoE−/−* mice, OI mice with Ang II infusion; duration: 4 weeks	*Col1a2+/G610C.ApoE−/−* mice, OI mice with AngII infusion were treated with 25 mg/kg Apc001PE twice a week for 4 weeks. This model was used to assess the cardiovascular risk Apc001PE, a Scl-Ab that targets only loop 3 of sclerostin, theorizing that the inhibition of the first 2 loops increases the cardiovascular risk. Apc001PE promoted the formation of the bone, without an increase in the cardiovascular risk.
Dattagupta et al. 2023 [68]	Case report: 52-year-old woman with type I OI; duration: 12 months	The patient received romosozumab, one dose of 210 mg monthly for 12 months, with improvements in BMD (10.3% in the spine and 5.4% in the right hip, *p* > 0.05) and no complications reported.

### 3.4. Recombinant Human Parathormone

(PTH) (teriparatide) is a potent osteoanabolic agent currently used in adult osteoporosis [69].

#### 3.4.1. Mechanism of Action

Teriparatide is a recombinant human PTH that increases the osteoblasts’ survival and their number in three ways: (1) increasing pro-osteoblastogenic factors like fibroblast growth factor 2 (FGF2) and insulin-like growth factor 1 (IGF1), using the upregulation of transcription in these factors; (2) the downregulation of wnt-antagonist sclerostin; and (3) it increases the expression of Runx2 (a transcription factor that is involved in the differentiation of osteoblasts). Teriparatide has the same effect as endogen PTH, increasing serum calcium levels and decreasing serum phosphate [69,70].

#### 3.4.2. Human Studies

In 2014, Orwoll et al. published a double-blind, placebo-controlled trial on 78 adults with OI over a period of 18 months. Forty of them were randomized in the placebo group and 38 in the teriparatide therapy one. The placebo group included patients with OI type I (27 patients), type III (7 patients) and type IV (5 patients), and the treatment group included patients with OI type I (24 patients), type III (7 patients), and type IV (7 patients). At the end of the study, 65 participants completed the protocol, but only 56 could be analyzed (27 in the placebo group and 29 in the treatment group). The treatment group received 20 μg of teriparatide daily for 18 months. The BMD change was higher in the treatment group, with a decline in the vertebral bone mineral density (vBMD) in the placebo group and a considerable increase in the teriparatide group (–4.7% ± 5.7% vs. 18.3% ± 5.9% change; *p* < 0.05). These changes were significant for OI type I (4.5% ± 7.3% vs. –5.5% ± 7.3% change; *p* = 0.008), but the authors did not find a statistically significant change in types III and IV compared with the placebo group. The changes in LsBMD were also significant in the type I treatment group (*p* < 0.001). There was no difference in the fracture rates reported by the patients. The study conclusions are that the better response occurred in patients with a less severe type. No complications were reported [71].

In 2017, Leali et al. published a multicenter, randomized, double-blind prospective study of teriparatide (group A) vs. neridronate (group B) on 94 patients with OI type I over 24 months, with a randomization rate of 1:1. Group A received 20 μg of teriparatide daily and group B received 100 mg of neridronate every 3 months, both for 24 months. They reported a better BMD in the teriparatide group (mean change 5.1% vs. 1.6%, *p* < 0.001). Using the Short Form-8 health survey score (SF-8 score) to assess the quality of life, and even though an improvement was reported in both groups, better results are seen in group A. The fracture rate was lower in the group with teriparatide, but the difference was not statistically significant (*p* = 0.1). No complications were reported. It was concluded that the teriparatide group showed better results than the neridronate one [72].

In November 2023, Hald et al. submitted a protocol for a randomized trial (ISRCTN15313991) of using teriparatide daily for 2 years, followed by a dose of zoledronic acid in adults with OI, but the results are not yet available [73].

We found only three studies in the literature that included this therapy usage for OI. Despite good results, teriparatide was effective only for mild forms of OI and was tested only in adults.

### 3.5. Anti-Transforming Growth Factor βeta (TGF-β) Antibodies

#### 3.5.1. Mechanism of Action

TGF-β acts as a coordinator of osteoclasts and osteoblasts, and its excessive signaling is associated with a decrease in bone mass and increased bone fragility in OI; this last statement has been demonstrated by Grafe et al. in OI mouse models, where they found excessive TGF-β signaling. They also used a murine monoclonal antibody, 1D11, as anti TGF-β treatment, resulting in a correction of the bone phenotype [74,75,76].

#### 3.5.2. Human Studies

In 2022, Song et al. published a study about the use of TGB-β in OI. They first collected bone fragments from 10 children with OI type III and 4 non-OI children. The OI bone presented a disorganized Haversian system and increased TGF-β signaling. A phase 1 dose-escalating clinical trial (NCT03064074) included eight adults with OI for 6 months and evaluated the safety of an anti-TGF-β monoclonal human antibody (Fresolimumab), an antibody that neutralizes all three TGF-β homodimers. The patients were divided into two groups of four participants each, who received a different dose of Fresolimumab (a single dose of 1 mg/kg or 4 mg/kg). In the first group (1 mg/kg), one patient with OI type III, two patients with type IV, and one with type VIII OI were included. In the second group (4 mg/kg), one patient with OI type III and three with type IV OI were included. Both groups were followed for 6 months. A decrease in Osteocalcin (Ocn) was observed significantly more in the group with 4 mg/kg (*p* = 0.00045) without significant changes in CTX and Procollagen type 1 N-terminal propeptide (P1NP) between the two groups. In the low-dose group, the ones with type IV OI had a robust increase in LsBMD; the one with OI type III reported a drop in the BMD, while in the one with OI type VIII, no changes in the BMD were noticed. In high-dose group 2, of the ones with OI type IV, an increase in the BMD was shown, while the one with OI type III had a femur fracture, with difficulty in evaluation. Nine adverse events were reported, possibly related to the medication (two patients in the low-dose group and seven in the high-dose group). The conclusion was that this therapy was beneficial for those with OI type IV and could be a potential treatment [77].

Only one human phase 1 study is available to date, and it was conducted on the adult population. Beneficial effects on BMD were reported only for type IV OI.

### 3.6. Genes Therapy (Table 5)

#### 3.6.1. Mechanism of Action

As OI is a connective tissue disorder very often caused by dominant mutations in the genes *COL1A1* and *COL1A2*, gene silencing through RNA interference is a promising field. Interfering RNA (iRNA) was designed to target the allele carrying the mutations *COL1A1* and *COL1A2* in bone cells. But more than 800 different mutations are described, and it is quite impossible to create iRNA against each mutation; however, by developing small interfering RNAs (siRNA) against common polymorphic variations, it would be possible to silence the mutation wherever the mutation is located on the allele [78].

#### 3.6.2. Animal Studies

Rousseau et al. published, in 2013, a study in which they used iRNA for successful *Col1a1* silencing in an OI mouse model [79].

#### 3.6.3. Cell Studies

*COL1A1* and *COL1A2* mutations represent most cases of OI, but more than 20 other genes are associated with OI. There are various gene-targeted therapies: suppression of harmful transcripts, increased expression of healthy alleles, or gene repair [80].

In 1996, Wang and Marini published a case of antisense oligodeoxynucleotides used to selectively suppress the mutant type I collagen in fibroblasts from a patient with OI type IV. Suppression of the mutant message was conducted, but the suppression achieved was insufficient for clinical intervention [81].

In 2004, Millington-Ward published a study in which iRNA was used to downgrade the expression of *COL1A1* in mesenchymal progenitor cells (MPCs), with good results but allele specificity [82].

Chamberlain et al. published, in 2004, an article about the adeno-associated virus vectors (AAV-*COLe1INpA* gene targeting vector) in MSCs in two patients with OI with an improvement in collagen stability. In 2008, another study by Chamberlain was published about the treatment with MSCs to disrupt *COL1A2* with AAV in patients with OI. The cells produced normal type I procollagen [83,84].

In 2008, Lindahl et al. used siRNA to successfully silence *COL1A2* in bone cells from OI individuals. In 2013, Lindahl et al. published another study in which they used silencing by iRNAi in *COL1A1* and *COL1A2* genes in bone cells in patients with OI. The average mRNA levels from both genes were successfully significantly reduced [85,86].

The results look very promising in this kind of therapy, but all human studies are in vitro.

**Table 5:** Studies of gene therapy in OI.

Reference	Methods	Comments
Wang and Marini, 1996 [81]	Cell study: fibroblasts from a patient with OI type IV (*COL1A2* mutation heterozygous dominant)	The suppression of the mutant message is realized, but it was insufficient for clinical intervention (~50% suppression of the mutant chain).
Millington-Ward et al., 2004 [82]	Cell study: MPCs with *COL1A1* heterozygous dominant mutation	The mutation is downgraded successfully (up to 85%), but the study concluded the need for allele specificity.
Chamberlain et al., 2004 [83]	Cell study: MSCs from 2 individuals with OI and *COL1A1* heterozygous dominant mutation	The AAV-*COLe1INpA* gene-targeting vector was used to disrupt the exon 1 of the *COL1A1* chromosome. The targeted cells showed an improvement in collagen stability, and the produced fibrils were closer to the wild-type mice, producing normal collagen.
Chamberlain et al., 2008 [84]	Cell study: MSCs from patients with OI heterozygous dominant *COL1A2* mutation	AAV, initially targeting exon 4, was used with unsuccessful results, so by targeting exon 2, the production of abnormal proα2(I) chains was eliminated, producing normal type I procollagen.
Lindahl et al., 2008 [85]	Cell study: bone cells from OI heterozygous dominant *COL1A2* mutation	siRNA could successfully silence *COL1A2* (0.3 µg siRNA dosing, 71%; 0.45 µg, 77%; 0.6 µg, 82%) in bone cells from OI individuals.
Lindahl et al., 2013 [86]	Cell study: bone cells from OI heterozygous dominant *COL1A1* and *COL1A2* mutations	Using siRNA, the average mRNA levels from both genes were successfully significantly reduced.
Rousseau et al., 2013 [79]	Animal in vitro and in vivo study: Brtl OI mouse (*Col1a1tm1.1 Jcm*, MGI: 2158863)	With one siRNA (F-Mut) used in vivo, there was 52% suppression of the mutant allele with only 14% of the normal allele with a ~ 40% decrease in the mutant protein.

### 3.7. 4-Phenylbutiric Acid (4-PBA) (Table 6)

#### 3.7.1. Mechanism of Action

The altered collagen products in OI are responsible for endoplasmic reticulum (ER) stress. The accumulation of misfolded proteins in the ER activates a specific response—the unfolded protein response (UPR)—that may initiate pro-apoptotic pathways. 4-PBA alleviates ER stress by helping to fold proteins into the ER and maintaining the homeostasis of the ER [87,88].

#### 3.7.2. Animal Studies

In 2017, Gioia et al. published a study that used a Zebrafish larvae mutant model called “Chihuahua” (*Chi/+*) that carries a substitution (G574D) in the α1 chain of type I collagen, validated as a model for OI. 4-PBA is a drug that, through a reduction in ER stress, stimulates collagen secretion and ameliorates the OI phenotype (bone mineralization), making it a promising candidate for the treatment of OI [89].

In 2022, Duran et al. examined the effect of 4-PBA on mouse models *Aga2+/−,* a model that manifests moderate and severe OI, reporting improvements in growth and bone resistance [90].

In 2022, Scheiber et al. published an animal study on OI mice treated with 4-PBA, showing a reduction in growth deficiency but without ameliorating bone fragility [91].

Daponte et al., in a study published in 2023, used two different Zebrafish models, Chihuahua (*Chi/+*) (dominant model) and *p3h1−/−* (recessive model), that lacks an enzyme, prolyl 3-hydroxylase (P3h1), proving that 4-PBA enhanced bone formation only in the recessive model [92].

#### 3.7.3. Cell Studies

Besio et al. conducted a study in 2018 treating human mutant fibroblasts from a skin biopsy of patients with OI with 4-PBA, showing a decrease in stress and apoptotic markers and an increase in general protein secretion in all treated cells [93].

In 2019, Takeyari et al. published a study using 4-PBA on human mutant fibroblasts, reporting ameliorated overglycosylation and the capability of calcification and diminishing the production of excessive collagen type I and its accumulation in fibroblasts [94].

Takeyari, in another study published in 2021 with 4-PBA in dermal fibroblasts from six patients with OI, noticed that the medication improves osteoblast mineralization, reduces ER stress, and normalizes the production of type I collagen [88].

**Table 6:** Studies of 4-PBA in OI.

Reference	Methods	Comments
Gioia et al. 2017 [89]	Animal study: Zebrafish larvae Chihuahua (*Chi/+*), model for OI. They had 6 groups of fish, 2 placebo groups (WT, Chihuahua) and 4 treated groups (WT, Chihuahua) with either 4-PBA or tauro-ursodeoxycholic acid (TUDCA); duration: 3.5 months	The authors found an enlargement of ER in fibroblasts and osteoblasts due to the mutant collagen retention. By reducing ER stress with 4-PBA, an amelioration of skeletal deformities and an increase in BMD was noticed in the treatment group with 4-PBA, so 4-PBA would be an effective treatment in OI trough reduction in ER stress.
Besio et al. 2018 [93]	Human cells, fibroblast from 10 patients OI type II and III (5 with mutations in *COL1A1* and 5 in *COL1A2*); duration: 15 h	In treated cells with mutations α2-G697C and α2-G745C, a decrease in stress and apoptotic markers are observed, and an increase in general protein secretion in all treated cells.
Takeyari et al. 2019 [94]	Human cells: fibroblasts from 6 OI patients; duration: not specified	4-PBA improved overglycosylation, the capability of calcification, and decreased the production of excessive collagen type I and its accumulation in fibroblasts.
Takeyari et al. 2021 [88]	Human cells: fibroblasts from 6 OI patients; duration: 28 days	Improvement in osteoblast mineralization reduced ER stress and normalization of the production of type I collagen was observed.
Duran et al. 2022 [90]	Animal study: 2-month-old *Aga2+/−* mice, model for moderately severe OI; duration: 5 weeks	The mice were treated with 50 mg/day 4-PBA, and a reduction in ER stress and better bone quality in vivo was noticed, with an increase in growth and bone resistance in the study’s drug group.
Scheiber et al. 2022 [91]	Animal study: 3-week-old OI model mice *Col1a2(+/G610C)* Four groups: - 2 Placebo WT and the model - 2 Treatment WT and the model; duration: 10 days	The treated mice displayed a reduction in growth deficiency, an improvement in the femur length, an improvement in BV/TV, trabecular BMD, and thickness, but without an amelioration in bone fragility, with no significant effect on biomechanical proprieties. No complications were reported.
Daponte et al. 2023 [92]	Animal study: Zebrafish, 2 models, a dominant one (Chihuahua” (*Chi/+*)), and a recessive one (*p3h1−/−*). They had 4 groups of fish: 2 placebo groups (model WT/model Chihuahua) and 2 treatment groups (model WT/ model Chihuahua); duration: 14 days	The study was conducted on 5 groups of fish: 3 placebo groups (wild-type) and the 2 models; 2 treatment groups of the 2 models. They amputated the caudal fins and allowed them to grow back to investigate the synthesis of collagen and bone differentiation with a beneficial effect of 4-PBA in the recessive model. No complications were reported.

These studies are reporting a beneficial effect on the production of collagen type I, but there are no in vivo studies on humans with OI.

### 3.8. Inhibition of Eukaryotic Translation Initiation Factor 2 (eIF2α) Phosphatase Enzymes (Salubrinal)

#### 3.8.1. Mechanism of Action

Salubrinal is an agent that is a specific inhibitor of eIF2α phosphatase enzymes. Elevated phosphorylation of this factor stimulates bone formation and reduces bone resorption. eIF2α stimulates bone formation by activating transcription factor 4 (ATF4) and, by phosphorylation of eIF2α, reduces the translation efficiency of most proteins, except for a limited number, one of them being ATF4. Another effect of Salubrinal is the suppression of RANKL activation [95,96,97].

#### 3.8.2. Murine Studies

The only study discovered was an animal study with a duration of 2 months, published by Takigawa in 2016, on 6-week-old Oim mice (*þ/; B6C3Fe a/a-Col1a2OIM/J*). The study had three groups: the control one with wild-type mice, group 2 (placebo) with Oim mice that received a placebo, and group 3 (treatment) with Oim mice that received daily Salubrinal at 2 mg/kg for 2 months and then followed for another 2 months. Compared with the placebo group, there was no difference in BMD, but they reported improvements in the mechanical proprieties compared with the placebo group. The femur stiffness (N/mm) and elastic module (GPa) were undistinguishable when compared with the control group. No complications were reported [98].

#### 3.8.3. Human Studies

We found no studies of Salubrinal and humans with OI.

We only found one study of this therapy in OI, and that study was on animals. More research is needed to analyze the effects of Salubrinal in OI.

## 4. Limits

A high number of studies are on animals or cell studies. Some medications do not include in vivo studies or studies in humans with OI, making it difficult to assess the efficacity of these therapies. More studies with a larger lot of subjects need to be researched further to evaluate the complications of each therapy. The treatments could not be compared for the following reasons: 1. Some therapy studies are in few numbers or are not human studies. 2. The heterogeneity of the disease is very complex, with multiple genes involved or clinical types of disease. 3. The age of the patients is different in human studies, with some involving adults, children, or both. More research is needed that involves each category of age.

However, this review’s objective was not to compare the medication but to highlight and describe other therapies aside from bisphosphonate that are now researched for OI patients.

## 5. Conclusions

Osteogenesis imperfecta is a rare genetic disorder of connective tissues with skeletal dysplasia and bone fragility. The actual therapies for OI are not efficient enough, and many research studies focused on the development of much-targeted therapies to improve low bone mineral density but also inherent bone fragility in the affected individuals, influencing the outcome. Other new potential therapies for OI, such as mesenchymal stem cells, anti-RANKL antibodies, sclerostin inhibitory antibodies, recombinant human parathormone, TGF beta inhibition, and gene therapy, showed promising results in vitro and in vivo in animal models or human practice, but they need to be researched furthermore.
